# Application of Artificial Neural Networks for Accurate Determination of the Complex Permittivity of Biological Tissue

**DOI:** 10.3390/s20164640

**Published:** 2020-08-18

**Authors:** Julian Bonello, Andrea Demarco, Iman Farhat, Lourdes Farrugia, Charles V. Sammut

**Affiliations:** 1 Department of Physics, Faculty of Science, University of Malta, MSD 2080 Msida, Malta; andrea.demarco@um.edu.mt (A.D.); iman.farhat@um.edu.mt (I.F.); lourdes.farrugia@um.edu.mt (L.F.); charles.v.sammut@um.edu.mt (C.V.S.); 2 Institute of Space Sciences and Astronomy, University of Malta, MSD 2080 Msida, Malta

**Keywords:** complex permittivity measurement, artificial neural network, in-vivo, biological samples

## Abstract

Medical devices making use of radio frequency (RF) and microwave (MW) fields have been studied as alternatives to existing diagnostic and therapeutic modalities since they offer several advantages. However, the lack of accurate knowledge of the complex permittivity of different biological tissues continues to hinder progress in of these technologies. The most convenient and popular measurement method used to determine the complex permittivity of biological tissues is the open-ended coaxial line, in combination with a vector network analyser (VNA) to measure the reflection coefficient (S11) which is then converted to the corresponding tissue permittivity using either full-wave analysis or through the use of equivalent circuit models. This paper proposes an innovative method of using artificial neural networks (ANN) to convert measured S11 to tissue permittivity, circumventing the requirement of extending the VNA measurement plane to the coaxial line open end. The conventional three-step calibration technique used with coaxial open-ended probes lacks repeatability, unless applied with extreme care by experienced persons, and is not adaptable to alternative sensor antenna configurations necessitated by many potential diagnostic and monitoring applications. The method being proposed does not require calibration at the tip of the probe, thus simplifying the measurement procedure while allowing arbitrary sensor design, and was experimentally validated using S11 measurements and the corresponding complex permittivity of 60 standard liquid and 42 porcine tissue samples. Following ANN training, validation and testing, we obtained a prediction accuracy of 5% for the complex permittivity.

## 1. Introduction

The complex permittivity of materials underlies their interaction with electromagnetic (EM) fields and provides information on how EM energy is absorbed and dissipated. This information is particularly important for many fields of research and development, and in recent years, considerable efforts were made to address gaps in knowledge identified by the medical technology industry. Accurate knowledge of the complex permittivity of human tissues is crucial in the design and development of diagnostic and therapeutic EM medical devices which are gaining increasing attention. This is because RF and MW radiation is non-ionising and has potential application in developing non- or minimally invasive theranostic devices. Examples of such applications are MW hyperthermia, ablation and imaging being investigated for diagnosis and treatment of a number of clinical conditions, such as early stroke detection and cancer [1,2,3,4,5,6]. Accurate knowledge of the dielectric properties of human tissue is not only required in the device design phase but is also essential for the creation and use of pre-treatment planning protocols, involving patient-specific 3-D EM field simulations. This is important in the case of microwave ablation, where an accurate estimate of the specific absorption rate (SAR) at various points around the MW applicator allows for the adjustment of input power, frequency, duration and insertion point in order to optimise the treatment outcome.

### Measurement Methods

Numerous methods exist for measuring the dielectric properties of different materials. Some of the most common include cavity perturbation, transmission line, tetrapolar impedance and open-ended coaxial probes [7,8].

**Figure 1:**
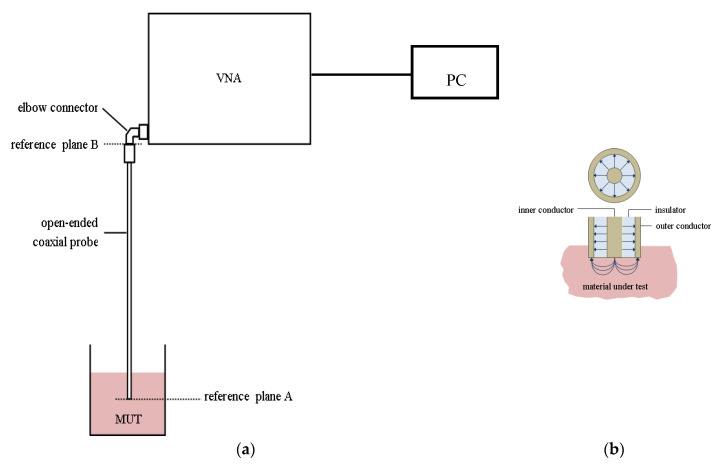
Open-ended coaxial probe technique: (**a**) Schematic of the measurement set-up, showing the vector network analyser (VNA) test port connected to the coaxial probe via an elbow connector, and the material under test (MUT); (**b**) cross-sections of the coaxial probe open end, showing the electric field configuration inside the coaxial line and the fringing fields protruding into the MUT.

The open-ended coaxial probe method is widely used for measuring the dielectric properties of liquids and semi-solids, such as biological tissues [7,9,10,11,12,13]. A typical setup used to perform dielectric measurements is shown in Figure 1b and includes an open-ended coaxial probe, connected to a VNA. The open tip of the coaxial transmission line is either embedded in or placed in contact with the material under test (MUT) such that the fringing fields emanating from the open tip reside within the MUT, as shown in Figure 1b. The VNA test port emits a MW signal swept over a pre-selected frequency range and receives the reflection from the probe tip. It then computes the reflection coefficient (S11) at the pre-selected frequency points and the corresponding complex permittivity is computed [10,11,12,13,14,15].

Several publications have reported different methods to calculate the complex permittivity from the measured S11 [15,16,17,18,19]. Reference [20] compares two methods for converting S11 to complex permittivity; one is based on a lumped-element equivalent circuit while the other implements a full-wave analysis, requiring solution of the theoretical model for the fringing EM fields, which is referred to as the forward problem. Once the forward problem is formulated, the inverse problem is solved iteratively to determine the complex permittivity of the MUT from the measured S11 [21]. This is computationally intensive and the inverse problem can be ill-posed, meaning that the solution does not always converge. On the other hand, the lumped-element equivalent circuit model provides a simpler approach by modelling the fringing fields at the tip of the coaxial probe through lumped equivalent circuits [22,23,24,25,26,27,28,29,30,31], and has become more popular over the years. The parameters for the equivalent circuits can be calculated from measurements on samples whose complex permittivity is accurately known, such as deionised water and NaCl solutions of precise concentration. Once these parameters are obtained for the standard liquids, they can be used to determine unknown dielectric parameters for other materials.

Whilst these methods are not as computationally intensive as the full-wave analysis, they depend on the initial measurements of three standard liquids [32]. This means that the computed complex permittivity is highly dependent on the initial three reflection measurements and minor errors could propagate significantly to the computed complex permittivity of the MUT.

Recently, commercially available open-ended coaxial lines have gained popularity for measurements conducted and reported by various research groups. Some manufacturers provide dielectric measurement kits, along with software to compute the complex permittivity from reflection coefficients measured by a VNA. The commercial software is unfortunately a black-box, providing little or no control on the computational algorithms used and present compatibility issues when using hardware and software from different manufacturers. Moreover, these kits require a three-step calibration at reference plane A in Figure 1; open-circuit, short circuit and a standard liquid which is usually deionised water [10,11,12,13]. This procedure requires skill and experience to guarantee acceptable repeatability, especially when terminating the coaxial open end with a short circuit so as to ensure good and uniform contact with the conducting surface.

In this paper, we propose an alternative method which overcomes such limitations by using an Artificial Neural Network (ANN) to convert measured reflection coefficients to complex permittivity. With this method, S11 measurements can be performed with any arbitrary VNA-probe configuration and then converted to the complex permittivity of a MUT. In our proposed method, any in-house designed probe can be used since the determination of the complex permittivity depends solely on the measurement of the reflection coefficient, without requiring VNA calibration extension to the measurement plane at the probe-material interface. This provides complete flexibility in the choice of hardware and experimental setup.

The reflection coefficient *Γ* (S11) at the boundary separating two semi-infinite regions with complex permittivities $\varepsilon_{1}$ and $\varepsilon_{2}$, assuming non-magnetic materials of finite conductivity, is obtained by considering the tangential electric and magnetic intensity components which are continuous across the boundary, leading to

(1) $$\Gamma =\frac{Z_{1}-Z_{2}}{Z_{1}+Z_{2}},$$

where $Z_{1}$ and $Z_{2}$ are respectively the wave impedances in medium 1, from where the wave is incident on the boundary, and medium 2, such that

(2) $$Z_{1}=\sqrt{\frac{\mu_{0}}{\varepsilon_{1}}}\;;\;Z_{2}=\sqrt{\frac{\mu_{0}}{\varepsilon_{2}}}\;\;,$$

where $\mu_{0}$ is the free space permeability since the material media are assumed non-magnetic. Thus, substituting for $Z_{1}$ and $Z_{2}$,

(3) $$\Gamma =\frac{\sqrt{\varepsilon_{2}}-\sqrt{\varepsilon_{1}}}{\sqrt{\varepsilon_{2}}+\sqrt{\varepsilon_{1}}}\;.$$

**Figure 2:**
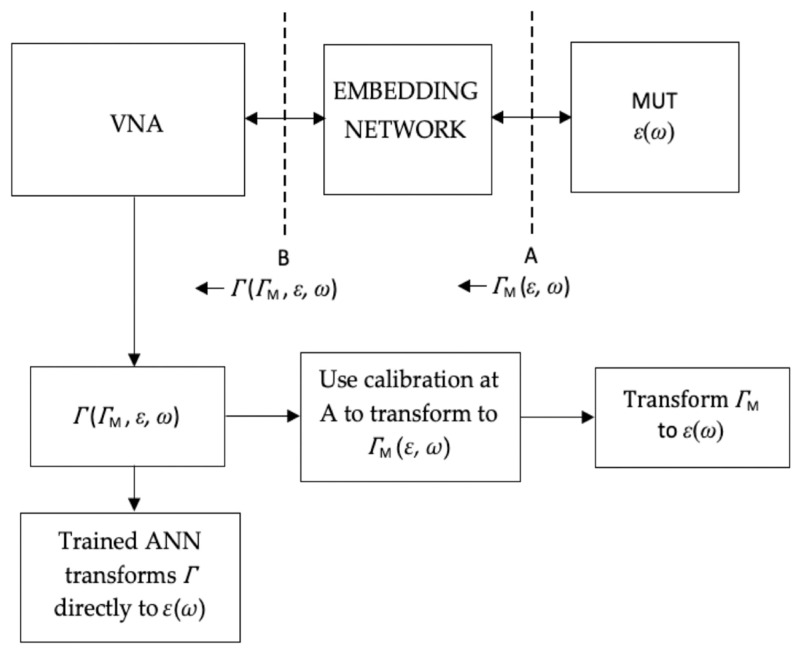
A schematic of a typical dielectric measurement setup, showing the measurement and transformation process to obtain the permittivity of the MUT. Also shown is the proposed ANN technique, requiring only calibration at the VNA test port (reference plane B in Figure 1). $\Gamma_{M}$ and Γ are respectively the reflection coefficients at the probe-material interface (reference plane A) and the VNA test port (reference plane B)

Equation (3) is non-linear and implies that *Γ* can be transformed to $\varepsilon_{2}$ at all frequencies, providing *ε*_1_ is known. Clearly, the fringing fields at the antenna/MUT interface have non-tangential components that are not considered in the above simple analysis. However, the one-to-one relationship between *Γ* and $\varepsilon_{2}$ still exists. Figure 2 describes conceptually how the conventional open-ended coaxial technique obtains $\varepsilon_{2}$ for a MUT, and compares this process with the proposed ANN conversion approach.

Training of the ANN was based on a large number of measurements (>50), thus reducing reliance on precise calibration and the subsequent validation required by equivalent circuit methods [10,11,12,13]. The proposed method can be reused, without the necessity of repeated measurements on all samples, as long as no changes occur in the measurement setup. In this work, we propose a shift of the calibration plane to the VNA test port (reference plane B in Figure 1), using only standard and repeatable coaxial open and short circuits as well as a matched load, which greatly simplifies the procedure and facilitates measurements in challenging scenarios such as sterile environments where in-vivo measurements are sought. Our alternative conversion technique is suitable for wideband and, even more so. for single frequency measurements.

## 2. Materials and Methods

In this work, a setup similar to that described in was employed, consisting of a VNA (Rhode & Schwarz, Germany, ZVA-50) connected to an open-ended coaxial probe (Keysight Technologies, USA, Slim-Form probe (85070E)) via an elbow connector. This arrangement enabled the probe assembly to be held fixed while the MUT is brought into contact with the probe tip by raising the container on a lab jack, thus avoiding the introduction of phase error caused by disturbing the measurement assembly.

Wideband measurements were conducted from 500 MHz to 5 GHz, with a total of 501 measurement points distributed linearly for each test sample. The temperature of the sample under test was constantly monitored with a Luxtron FOT lab kit and the recorded temperatures varied between 22 °C and 25 °C.

The data collection was organised in two phases. The first phase consisted of the collection of the complex permittivity, referred to as output data, and the second phase focused on the measurements of the reflection coefficient, referred to as the input data. The output data was measured following a rigorous calibration and validation of the measurement system. In this case, a three-step calibration was conducted: (1) the tip radiating in free space (open), (2) the tip shorted at its end (short) and (3) with the probe tip immersed in a liquid of known dielectric properties at a known temperature (load). For this study, deionised water at 22 °C was used as the load. Following calibration, validation was performed where the complex permittivity of a 0.1 M NaCl solution was measured and compared to values published in Peyman et al. [33]. Once, the validation step was completed, the complex permittivity of the liquid samples was measured.

The second phase consisted of measuring the input data and this was done for two different calibration reference planes (Figure 1): A at the tip of the probe and B at the VNA test port. Calibration at reference plane B was a standard three-step procedure using a 2.4 mm open (Fairview Microwave SC2040 [34]), short (Fairview Microwave SC2152 [35]) and a matched termination (Fairview Microwave ST5038 [36]).

Measurements were conducted on a total of 102 different material samples, as listed in Table 1, including a number of porcine fat and liver tissues. Various standard liquids were carefully selected so that their complex permittivity covered a wide range of values while the porcine tissues were used to assess the influence of expectedly wide inter-sample permittivity variation on our measurement technique and its efficacy.

Individual liquid samples were prepared as indicated above while the porcine liver and fat tissues from four different animals were acquired from the public abattoir in Marsa, Malta, within two hours of animal slaughter. These were preserved for transport in closed plastic containers in order to minimise tissue dehydration. Once the tissues were inside the laboratory they were sectioned into individual liver and fat samples, each of volume approximately 60 cm^3^, in preparation for measurements that took place within 30 min following sectioning.

The uncertainty in the measured output data (Table 2) was calculated using the method proposed by Peyman et al. [37] which was also adopted in [10,11,12,13]. A component of the combined uncertainty reported in this table is a comparison to literature (0.1 NaCl in this case), as specified by Peyman et al. in [37]. In addition to this, the uncertainty also considers the standard deviation in the measured complex permittivity on each particular sample.

### Implementation of the ANN

**Figure 3:**
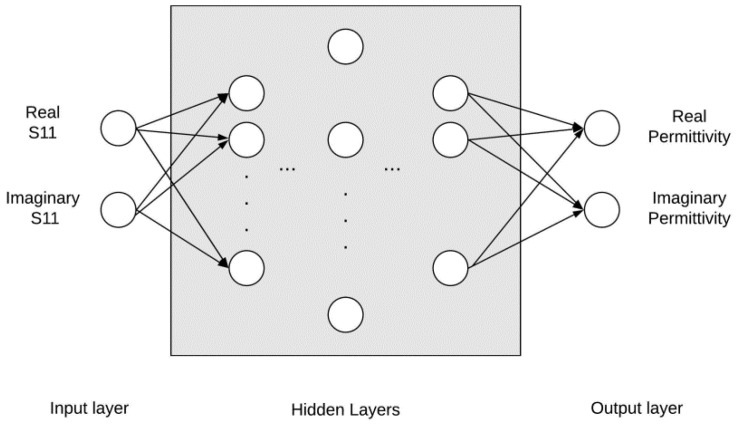
The ANN architecture showing the input layer which is composed of both the real and imaginary S11 as inputs. They grey box encapsulates the five hidden layers which have, respectively from left to right, 200, 400, 600, 400, 200 nodes. The output layer is made up of the real and imaginary permittivity as two distinct outputs.

A typical ANN configuration is made up of a number of nodes arranged into layers, as shown in Figure 3, with each node in one layer being linked to a node in the adjacent layer (moving from left to right), each link having a weight associated with it. The computed value for the complex permittivity at the output layer is obtained by taking the summation of all possible combinations between the input and output layers. Iteration is performed every time this value is generated. After the successful completion of an iteration cycle, the loss value is calculated and the weights of the network are optimised through back-propagation with the aim of reducing the loss value.

**Figure 4:**
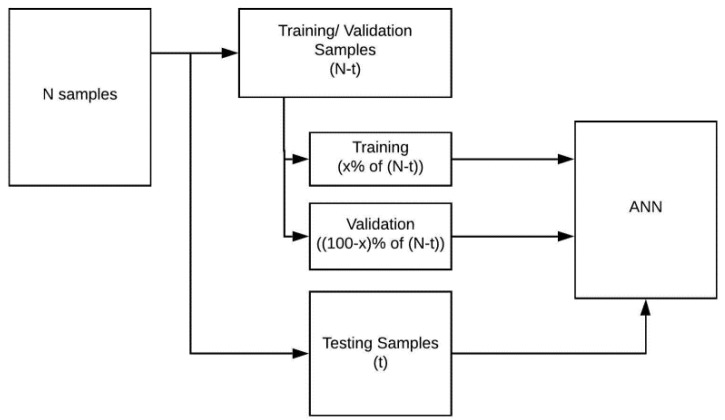
A schematic of the data organisation used in the ANN. In this study N = 102, representing all tested samples, and x varied between 50% and 90%.

Following standard implementations, the input and corresponding output data (N = 102) were divided into two sets, one for training and the other for testing, as shown in Figure 4. The former was used to train and validate the ANN while the latter was used to assess the accuracy of the trained model. The data forming part of the training set (N-t) was randomly split into two; a specified fraction (x% of (N-t)) being used for training and the remaining data used for validation. The fraction x varied between 50% and 90%, as described in Section 3.

During the training process, the weights within the ANN were adjusted so as to minimise the final loss value. The latter refers to the accuracy of the model and is obtained by calculating the mean square of the difference between the computed value using the ANN and the expected output which in our case was the complex permittivity measured by the conventional open-ended coaxial probe technique. The main aim was to derive an ANN with a loss value converging to a minimum within a reasonable number of iterations. The model was considered to have reached convergence when the variations in the loss value did not exceed the set tolerance for successive 10 iterations. Different configurations of the ANN were tested to allow for model improvement by reducing loss values until an optimum was achieved, thus determining the final ANN configuration to be implemented.

**Figure 5:**
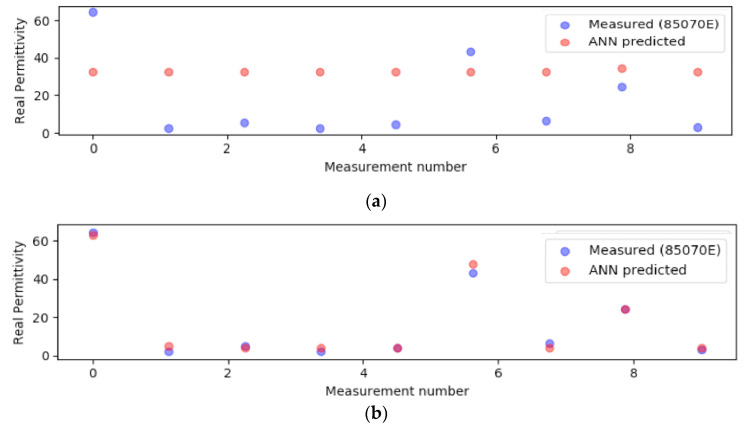
Two example plots showing model performance on randomly selected validation data: (**a**) a model in which the parameters were not refined; (**b**) the same model with refined parameters, showing vastly improved performance.

Once the model converged on the training set, the validation data set was used to assess the quality of learning and to predict model performance [38]. The scope of the validation procedure is to refine the ANN architecture and parameters, as described in the following paragraphs. For example, Figure 5a,b compare the real part of the relative permittivity predicted by the ANN at 2.45 GHz with the corresponding measured values, respectively in early training stages and after all training epochs had been performed. The points on Figure 5b converge remarkably well following this procedure.

**Figure 6:**
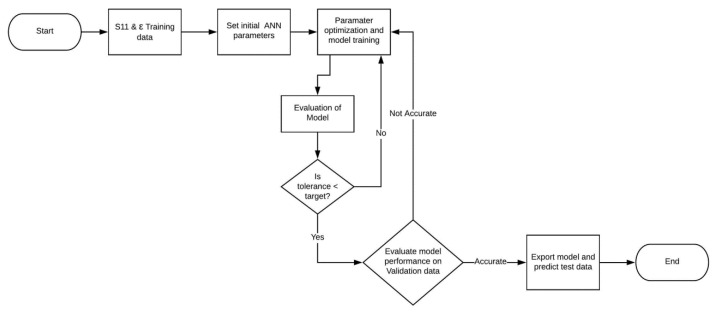
A schematic diagram showing training, validation and testing sequence of the ANN.

Finally, the test data was used to provide an unbiased evaluation of the final model and its performance. Figure 6 shows a schematic of the complete process, starting from the data input stage up to the testing of the ANN.

For both situations investigated (calibration at reference planes A and B), 4-fold cross validation for different training/validation/test data splits was performed. This means that the data appertaining to the training, validation and test sets were varied four times and the performance of the model was evaluated in each case. This process ensured that the performance was consistent and repeatable.

The training of the ANN was performed using a multilayer perceptron regressor (MLPRegressor) algorithm coded in Python [39]. Different configurations were tested and the best results were obtained with an architecture consisting of 5 successive layers, with 200, 400, 600, 400, 200 hidden nodes in each layer respectively (refer to Figure 3, starting from left). The activation function used was the rectified linear unit (ReLU), with the solver (optimisation algorithm) selected being Adam. The maximum iterations permitted were 500,000 while the tolerance was set to $1.0\times 10^{-8}$, with a learning rate of $5\times 10^{-5}$.

The S11 measurements from each sample constitute the ANN input at each of the 501 frequency points. The ANN accepts two inputs, the real and imaginary parts of the measured S11 at each frequency point, which are then passed on to the hidden layers for training. The ANN then outputs separate values for the corresponding real and imaginary parts of the complex permittivity at each of the 501 frequency points; this architecture is shown in Figure 3.

## 3. Results

This section presents and analyses the results obtained once the ANN parameters were tuned and optimised. The results are presented in two sub-sections corresponding to calibrations performed at reference planes A and B, as detailed in Section 2. We also evaluated the performance of the ANN at 2.45 GHz, this being a widely used frequency for MW ablation, and investigated the effect of adding noise to the input data to assess the model’s robustness. Finally, we discuss the effect on model accuracy of reducing the training data set from 91 to 45 data points.

### 3.1. Calibration Performed at the Tip of the Probe—Reference Plane A

#### Analysis of Broadband Measurement and Predicted Results

**Figure 7:**
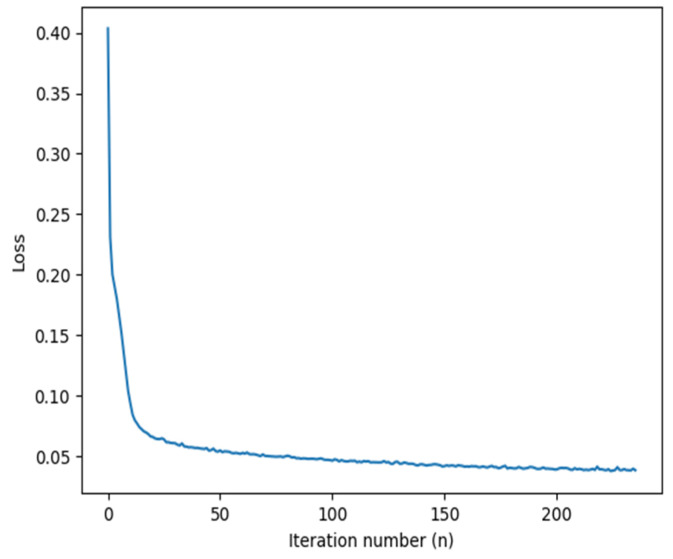
A plot showing variation in the loss value as a function of number of iterations for the model generated. Calibration point: reference plane A (probe tip); data predicted: dielectric permittivity spectrum.

Figure 7 represents the variation of the loss function with the number of iterations, starting from an initial value of 0.410 and, after 236 iterations, converging to 0.038. The results obtained on the validation data showed good agreement between the predicted results and the actual measurements. The performance of the model was further tested on the test data set, the results of which are discussed next.

**Figure 8:**
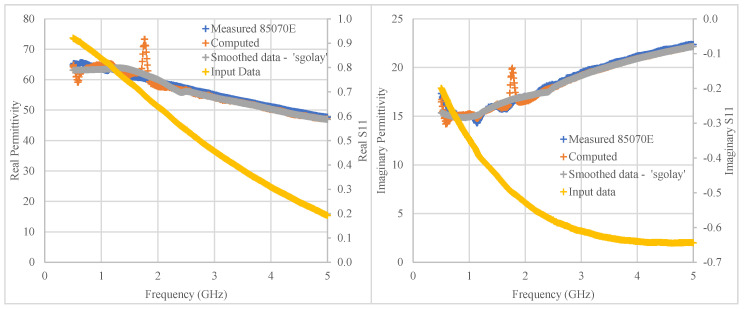
Plots showing the real and imaginary parts of the permittivity of 30 mL of 0.1 M NaCl + 20 g glucose from 0.5 to 5 GHz. The values measured with the slim-form probe appear in blue, the corresponding ANN-predicted values are marked in orange while the grey data points show the corresponding predicted values following smoothing with Sgolay. The corresponding S11 measurements are shown in yellow for comparison. Calibration point: reference plane A (probe tip). Note: The computed (ANN predicted) values for both real and imaginary parts of the permittivity are a function of both parts of S11 at each frequency.

In this analysis, all the measured data points over the frequency range 500 MHz to 5 GHz (16,533) were pooled to train and validate the model, except for one set of 501 frequency points for 30 mL of 0.1 M NaCl + 20 g glucose that were used for testing over this frequency range, as shown in Figure 8. While the ANN can only be made to predict the complex permittivity at trained frequencies, should any value be required at intermediate frequency points, these can be obtained by interpolating between the two adjacent points at higher and lower frequencies. The training set was split with a ratio of 0.1, meaning 90% was used for training while the remaining 10% was used for model validation.

Following training and validation, the model’s performance was also evaluated using the test data set. The maximum and average percentage differences between the conventional measurements and the predicted values for the real part of the permittivity *ε*′ were respectively 17.60% and 2.09%, and 17.74% and 1.80% for the imaginary part, *ε*″, as shown Figure 8, where the measured permittivity is marked in blue and the corresponding ANN predictions are marked in orange. The latter were smoothed with the Savitzky-Golay filter (Sgolay, implemented in Matlab^®^) and results are marked in grey. The advantage of this filtering method is that it best retains the shape of the original system while smoothing the output ANN data, which is not readily achievable with a moving average filter [40].

Figure 8 indicates that the ANN performance was accurate above about 2 GHz, where the predicted values were within 1.7% of the corresponding measurements carried out with the slim form probe, following ANN output data smoothing. Below 2 GHz, the measured permittivity (especially the imaginary part) showed variations with frequency that were probably due to calibration issues and that had also been previously reported [10,41]. This could have been the reason for the relatively larger discrepancies between the measured and predicted values at the lower frequencies, where the maximum difference was recorded at 1.73 GHz 5.9 for *ε*′ and 3.5 for *ε*″.

### 3.2. Calibration Performed at VNA Test Port—Reference Plane B

In this section, we analyse the results obtained when shifting the calibration plane to the VNA test port, marked as reference plane B in Figure 1. Following calibration with standard open, short and matched terminations, initial tests were conducted with all the 16,533 points being considered over the frequency range. The ANN converged to a very low level of accuracy when evaluated on test data, resulting in an average percentage difference of above 15% from the measured permittivity. We therefore focused on a single frequency (2.45 GHz) to eliminate frequency dependent phase shifts and to simplify the problem. In this case, training and validation of the ANN was performed using measured S11 as input data. In order to evaluate the robustness of the method, we added Gaussian noise to the input data to simulate the effects of random fluctuations about the measured average of the reflection coefficient.

#### 3.2.1. Training of the ANN Using S11 Measured at the VNA Test Port

For the first test run, the ANN was trained on S11 measurements at the VNA test port (reference plane B) at 2.45 GHz. The training and testing data sets consisted respectively of 91 and 11 data points, representing all measurements obtained from all 102 samples. Therefore, 90% of the data was used for training while the remaining 10% was used for model validation.

The loss value was once again monitored throughout the learning process and a similar plot as in Figure 7 was obtained; in this case the loss factor converged to 0.00059084. The model’s performance was first evaluated with the validation data and since the points obtained indicated that the model was performing well, a more detailed analysis with the test data ensued.

**Figure 9:**
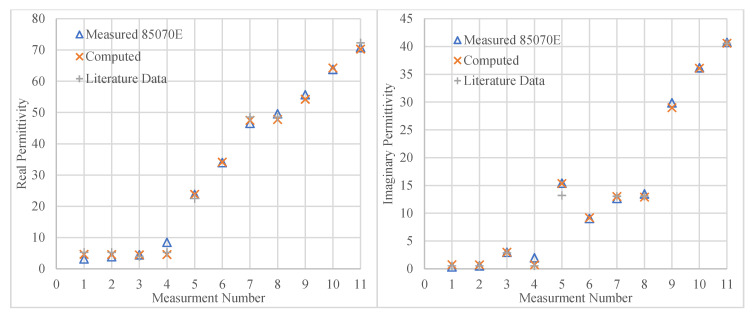
Plots comparing the actual measurement values (blue) with the predicted (orange) values for 11 measurement points. Probe used: Slim Form; Calibration point: VNA test port; data predicted: permittivity at 2.45 GHz. Available literature data: Fat [42], Propan-2-ol and Methanol [43], Liver [33], 0.5 M NaCl [44].

Figure 9 shows the computed complex permittivities using the ANN compared with those measured with the slim form probe for the 11 samples (test data), the list of samples measured is presented in Table 3. For comparison these plots also include points which are derived from published literature. The maximum difference in *ε*′ was 47.6%, corresponding to a difference of 1.50 between the measured and predicted values, when the measured value was 3.14. The calculated uncertainty in the measured *ε*′ was 2.6%. The average difference was 11.1% (1.04) between the predicted and measured values. For this test, the input data consisted of eleven points; four of which had *ε*′ < 8. Thus, even small discrepancies resulted in a considerable discrepancy, given that the mean was relatively small. For the remaining seven points, the maximum difference was 4.0%, corresponding to a difference of 1.90 in the *ε*′. The average difference across these points was 1.48%, indicating that for *ε*′ values above 10, the ANN computed the conversion accurately. In fact, the highest uncertainty values resulted for fat tissue, which has a relatively lower permittivity, while the difference for Isopropyl was calculated to be 0.22%. This result is understandable since when measuring fat tissue with the slim form probe, measurements vary significantly from point to point, even on the same sample, owing to the inherent heterogeneity of the tissue.

*ε*″ showed a higher maximum discrepancy between measured and predicted values (119%, corresponding to 0.41 for a measured value of 0.34. The calculated uncertainty in the measured *ε*″ was 1.2%. However, the model’s performance for *ε*″ > 3 was much better, where the maximum difference between measured and the predicted values was 4.26%, corresponding to an absolute difference of 0.57. The average difference between the measured and ANN predicted values across these latter points was 1.72% (0.30).

#### 3.2.2. Influence of Training Data Size on ANN Performance

The influence of the size of the training dataset on ANN performance was investigated by varying the ratio of the training to validation data. Initially, all 102 samples were considered with 90% of the data used for training and the remaining 10% for validation.

In this part of the analysis, the validation data was increased in steps of 10% up to 50%; the latter corresponding to 45 data points for training and 46 for validation. Table 4 summarises the results obtained for these different data configurations, illustrating that the calculated loss value increased with decreasing numbers of training data. The number of iterations required for the loss to be within the set tolerance (0.00000001) also increased. In fact, with 80% of the data used for training, the model converged within 1929 iterations while with 50% of the data available for training, 3076 iterations were required to converge to within the same tolerance.

Following model convergence with the training data sets, the performance was evaluated with the validation data and similar results were obtained. 

**Figure 10:**
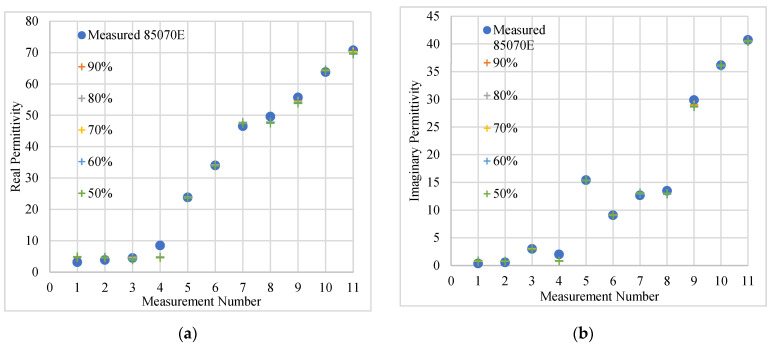
The computed and measured real (**a**) and imaginary part (**b**) of the complex permittivity for all testing data (eleven samples) at 2.45 GHz. The computed data was obtained by varying the ratio between the validation and training data. The percentages refer to the fraction of the data set used for training, the remainder being used for validation. The measured data was obtained using the Slim Form probe calibrated at reference plane A (probe tip) while the ANN input data was obtained with the calibration plane at the VNA test port (reference plane B in Figure 1).

The results obtained on the test data set for the different data configurations are presented in Figure 10, which shows the computed real (a) and imaginary (b) parts of the complex relative permittivity for the eleven data points considered in the testing set that correspond to the eleven samples used in this part of the study.

The measured and computed data for all samples are generally in good agreement for samples with *ε*′ > 10. The discrepancies tend to increase with decreasing fractions of the data set used for training, especially for the low permittivity samples (the first four points in Figure 10a)

Table 5 shows the maximum, minimum and average percentage differences between the complex permittivity measured with the Slim Form probe calibrated at the tip and that obtained by the ANN, following training based on S11 measured following VNA calibration at the test port. The maximum discrepancy (Δ max) in *ε*′ was 57.1%, equivalent to a difference of 1.79 between the measured (3.14) and computed values when the ANN was trained on 70% of the data. This did not vary significantly even when the model was trained with less data. In fact, with the ANN trained on 50% of the data, the discrepancy was 52.5%, corresponding to a difference of 1.75 between the computed and measured *ε*′.

When omitting the four measurements with low real permittivity (<8.48), the average discrepancy was calculated to be 1.48% in the best-case scenario (90% of the data available for training) and increased to 1.88% when 50% of the data was used for ANN training. This is below the 2.6% maximum combined uncertainty calculated for Slim Form probe measurements at 2.45 GHz (Table 2).

In general, there was good agreement when the measured and computed data was compared across all the samples with *ε*″ > 10. An increase in discrepancies was noted as the fraction of data used for validation increased, especially for low permittivity samples (the first four points in Figure 10b). The maximum discrepancy (Δ max) in *ε*″ registered was 152%, which is equivalent to a difference of 0.40 between the measured (0.34) and computed values when the ANN was trained on 50% of the data.

When the first four measurements (*ε*″ < 2.89) were omitted, the average discrepancy was calculated to be 1.72% in the best-case scenario (90% of the data available for training) and increased to 3.79% when 50% of the data was used for ANN training. This figure is lower than the 3.9% maximum combined uncertainty calculated for Slim Form probe measurements at 2.45 GHz (Table 2).

#### 3.2.3. Testing Robustness of the ANN by Adding Noise to the Raw Input Data

Any dielectric measurement has a certain degree of uncertainty associated with it and so far these have not been considered sufficiently. In order to investigate the effects of measurement uncertainty on the ANN predictions, noise was added to measured S11 input data to assess its effect on the predicted output. This is of particular importance since our technique is intended to be used for the extraction of the complex permittivity of biological tissues which are inherently heterogeneous, with properties that vary even between samples of the same tissue.

Measurement uncertainty is usually considered by quantifying and combining the uncertainties arising from sources of error such as drift, systematic and other random errors [12,45]. In order, to investigate the robustness of the ANN model in this regard, Gaussian noise was added to the measured input data by considering the average standard deviation (aSTD) for each frequency across measurements for the same material. The aSTD was considered as an estimate of the random errors in the average S11 measured by the VNA when calibrated at the test port (raw input data).

The data processing was performed in Matlab^®^ by generating a set of random data points with a normal distribution about the mean which was set equal to the average measured S11. Two sets of data were generated by randomly dispersing values within 1- and then 10-sigma about the measured S11. The first corresponds to ± 34.1% confidence interval, whilst the latter corresponds to ± 99.9%, depicting the worst-case scenario. The aSTD (1-sigma) for the real part of the relative permittivity was 0.002 while for the imaginary part, this was 0.04. In order to ensure reliability and repeatability of results, both sets of noisy data (ND) were generated twice and the analysis was split into two separate scenarios. In the first, the effect of adding noise to the training and validation data while not altering the test data was investigated, which doubled the amount of available training data. The second scenario introduced noise in both the training and testing data sets. The model converged for both sets of data with calculated loss values being all below 0.0005.

**Figure 11:**
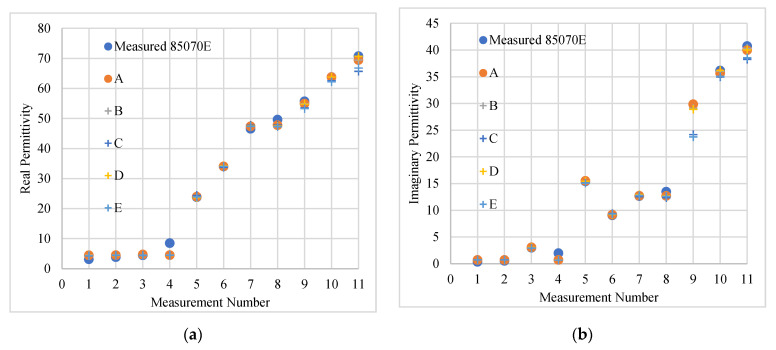
The computed and measured real (**a**) and imaginary part (**b**) of the complex permittivity for all testing data (eleven samples) at 2.45 GHz, considering both scenarios. Scenario 1 included noise added only to the training and validation data while Scenario 2 features noise in all training, validation and testing data. The measured data was obtained using the Slim Form probe and the input data (S11) was obtained with the VNA calibrated at the test port (reference plane B in Figure 1). Key: A—Raw data, B—Raw and noisy input data 1, C—Raw and noisy input data 1 with noisy test data, D—Raw and noisy input data 2, E—Raw and noisy input data 2 with noisy test data.

Figure 11 shows the variation of the real (a) and imaginary (b) parts of the relative permittivity of both the measured and computed values with the ANN for both scenarios with S11 obtained at 2.45 GHz. Both plots show good agreement between the two sets for all measurement points investigated and very minimal changes were observed when compared to the previous analysis using only raw input data.

Table 6 shows the maximum, minimum and average differences between the computed and measured values when considering the test data. The maximum difference for *ε*′ was obtained when noise was added to the input testing data. This resulted in a 92.8% difference, corresponding to an absolute difference of 4.08 in the *ε*′. When considering the seven points with *ε*′ > 10, the maximum difference was 4.55%, equivalent to an absolute 2.15 between the measured and the computed values. This is 1.95% higher than the uncertainty value in the measurement performed using the slim form probe (2.60%). However, this difference resulted when noise was added to the original measurement for the measured reflection coefficient.

The maximum difference for *ε*″ was 200%, equivalent to a difference of 4 between the computed and measured data. Since the absolute values of *ε*″ were as small as 0.34, small variations in this parameter yielded significantly large discrepancies.

The next set of results show the effect of adding noise which was tenfold larger when compared to the noise previously added. The variation in the loss as a function of number of iterations remained similar to the previous results and converged to a loss function value of below 0.0005, following sufficient iterations.

With 10-sigma noise added to the raw data, the overall predictions still remained accurate since the maximum and average differences for the *ε*′ did not increase when compared to the 1-sigma case. However, the average discrepancy for the *ε*″ increased by 5% in both sets of the noisy test input data, as shown in Figure 11 and Table 6. Considering the last 7 points (*ε*″ > 3), the discrepancy with both noisy testing data sets was below 4% for the *ε*′ and below 6% for *ε*″.

## 4. Conclusions

This work presents an innovative conversion method which accurately computes the complex permittivity from measured reflection coefficients by employing a suitably trained ANN. In our experiments, a commercial open-ended probe was used to facilitate comparison of results and validation of the technique with this widely-accepted dielectric measurement method. Two different calibration planes were considered where it was shown that for a broad frequency range, pre-measurement calibration at the probe tip produces results equivalent to those obtained by the standard method with S11 conversion achieved by software provided with a commercial measurement kit. In our experiments, the average difference between the measured and computed output data was 2.09% and 1.80%, respectively for the real and imaginary parts of the permittivity, well within the combined uncertainty resulting from the conventional coaxial open-ended probe measurement technique with calibration at the open end. Additionally, we found that model accuracy is enhanced by increasing the number of measurement points for frequencies below 1 GHz but this could have been due to issues arising from calibration or the commercial conversion model.

Spectral analysis of the complex permittivity with this technique requires a larger training set in comparison to narrowband conversion from measured S11 values with the VNA calibrated at the test port.

The ANN conversion technique at a single frequency of 2.45 GHz with VNA calibration at the test port produced very good agreement with conventional measurements when the ANN was fed with S11 values referenced to the calibration plane. Following ANN training at 2.45 GHz, the maximum difference between the measured and computed output data was 4.0% and 4.3%, respectively for the real and imaginary parts of the permittivity. This shows that the error associated with the ANN-extracted parameters is well within that stated by manufacturers of commercial probes (<10%) [45] and our own uncertainty estimates for this work.

In this study, we compare our ANN-based technique to the well-established and widely used open-ended coaxial probe measurement system, utilising commercially available probes and conversion software. The advantages of our system are not in terms of initial calibration time, since the ANN training requires more data acquisition. However, this technique allows arbitrary antenna configurations to be used for dielectric measurements, without having to carry out calibration at the antenna/material interface (Figure 2).

In the case of the simple geometry of a coaxial open-ended probe, the lumped element equivalent circuit of the antenna/material interface is conceptually simple, and the calibration technique establishes the element values. However, any alteration of the sensing antenna geometry requires a different equivalent circuit model and associated calibration procedure. Our proposed technique eliminates the need of devising an equivalent circuit and calibration procedure by transferring the measurement plane to the VNA test port. Apart from the universal standard open, short, and matched load at the VNA test port, the calibration procedure for any specific sensing antenna is then reduced effectively to a collection of a set of training measurements. Apart from widening the scope of development of novel antennas for RF and MW sensing, this technique solves inter-system compatibility issues, as in the case of using a probe and VNA from different manufacturers.

## Figures and Tables

**Table 1:** Test materials and respective number of samples. To the authors’ knowledge, there is no published information on the highlighted substances.

Test Material	Number of Samples
30 mL of 0.5 M NaCl	3
30 mL of 0.5 M NaCl + 2 mL TX-100	3
30 mL of 0.5 M NaCl + 4 mL TX-100	3
30 mL of 0.5 M NaCl + 6 mL TX-100	3
30 mL of 0.5 M NaCl + 8 mL TX-100	3
30 mL of 0.5 M NaCl + 11 mL TX-100	3
30 mL of 0.5 M NaCl + 4 mL TX-100 + 2 g glucose	3
30 mL of 0.5 M NaCl + 11 mL TX-100 + 2 g glucose	3
30 mL of 0.1 M NaCl + 20 g glucose	3
Acetone (107901Z-Brenntag)	3
Propan-2-ol (1376-Girelli Alcohol)	3
Methanol (20864. 320-VWR)	3
30 mL TX (TR04441000 – Scharlau)	6
15 mL TX + 15 mL DI water	6
15 mL TX + 20 mL DI water	6
15 mL Isopropyl alcohol + 15 mL DI water	3
27.5 mL Isopropyl alcohol + 15 mL DI water	3
Porcine liver (freshly excised)	21
Porcine fat (freshly excised)	21
Total	102

**Table 2:** Combined uncertainties in complex permittivity measurements performed on all materials with the Slim Form probe kit. *ε*′ and *ε*″ are respectively the real and imaginary parts of the permittivity.

Measurement Type	Δ *ε*′ [%]			Δ *ε*″ [%]		
	Max	Min	Mean	Max	Min	Mean
Broadband (0.5–5 GHz)	5.60 ^(1)^	0.31 ^(3)^	1.40	9.80 ^(2)^	0.03 ^(4)^	1.77
Single frequency (2.45 GHz)	2.60	0.60	1.20	3.90	1.00	2.50

^(1)^ at 4.6 GHz; ^(2)^ at 518 MHz; ^(3)^ at 527 MHz; ^(4)^ at 4.7 GHz.

**Table 3:** The measurement number and corresponding material name for the results shown Figure 9, with accompanying references to available relevant published data.

Measurement Number	Material Tested
1	Fat [42]
2	Fat [42]
3	Propan-2-ol [43]
4	Fat [42]
5	Methanol [43]
6	15 mL TX + 15 mL DI water
7	Liver [33]
8	Liver [33]
9	30 mL of 0.5 M NaCl + 11 mL TX-100 + 2 g glucose
10	30 mL of 0.5 M NaCl + 4 mL TX-100
11	30 mL of 0.5 M NaCl [44]

**Table 4:** The maximum number of iterations and corresponding loss values calculated using the last two iterations when training the ANN on different fractions of training data with respect to the validation data.

Data Fraction Used for Training [%]	Max Iteration Number	Final Loss Value
90	2099	0.00059
80	1929	0.00069
70	3081	0.00070
60	3243	0.00079
50	3076	0.00094

**Table 5:** Maximum, minimum and average differences between measured and predicted permittivity at 2.45 GHz with different ratios of training to validation data (Δ training). Probe used: Slim Form probe; Calibration point: VNA test port.

Δ Training [%]	Δ *ε*′ [%]			Δ *ε*″ [%]		
	Max	Min	Mean	Max	Min	Mean
90	47.6	0.15	11.1	119	0.16	21.0
80	54.0	0.29	12.5	126	0.07	21.8
70	57.1	0.48	12.4	140	0.01	23.4
60	55.8	0.47	12.8	150	0.27	25.0
50	52.6	0.56	13.2	152	0.07	25.2

**Table 6:** Maximum, minimum and average differences between measured and predicted permittivity at 2.45 GHz using raw input data (no added noise); Scenario 1 (noise added to training and validation data); Scenario 2 (noise added to training, validation and testing data).

ANN Results Compared to Slim Form Probe Measurements as Indicated	Δ *ε*′ [%]			Δ *ε*″ [%]		
	Max	Min	Mean	Max	Min	Mean
No added noise	88.5	0.07	13.5	184	0.01	24.7
Scenario 1	87.0	0.03	13.3	181	0.02	25.6
Scenario 1, 2nd run	92.8	0.78	13.2	202	0.15	29.7
Scenario 2	91.7	0.02	14.2	182	0.01	25.9
Scenario 2, 2nd run	91.4	0.12	14.0	198	0.86	29.3
